# From a PMT-based to a SiPM-based PET system: a study to define matched acquisition/reconstruction parameters and NEMA performance of the Biograph Vision 450

**DOI:** 10.1186/s40658-020-00323-w

**Published:** 2020-09-03

**Authors:** Thomas Carlier, Ludovic Ferrer, Maurizio Conti, Caroline Bodet-Milin, Caroline Rousseau, Yanic Bercier, Bernard Bendriem, Françoise Kraeber-Bodéré

**Affiliations:** 1 Nuclear Medicine Department, University Hospital of Nantes, Place Alexis Ricordeau, Nantes, France; 2 Université de Nantes, CNRS, INSERM, CRCINA, Nantes, France; 3 Nuclear Medicine Department, ICO, Boulevard Jacques Monod, Saint-Herblain, France; 4 Siemens Medical Solutions USA, Inc., Knoxville, TN USA

**Keywords:** Vision 450, NEMA, Noise, Optimization

## Abstract

**Background:**

The purpose of this work was to propose an approach based on noise measurement to adapt present clinical acquisition and reconstruction parameters adapted to a PMT-based system (Biograph mCT) to a SiPM-based system (Biograph Vision 450) sharing identical geometrical properties. The NEMA performance (NEMA) of the recently released Biograph Vision 450 PET/CT (Vision) was also derived.

**Methods:**

All measurements were conducted on Vision and Biograph mCT with TrueV (mCT). A full NEMA-based performance was derived for Vision only. The adaptation of acquisition and reconstruction parameters from mCT to Vision was done using the NEMA image quality phantom. The noise level reached using mCT was set as the reference value for six different numbers of net true coincidences. The noise level computed using Vision was matched to the reference noise level (within 0.01%) using a different reconstruction set-up to determine the potential reduction of count numbers for the same noise level.

**Results:**

Vision sensitivity was 9.1 kcps/MBq for a timing resolution of 213 ps at 5.3 kBq/mL. The NEMA-based CR for the 10-mm sphere was better than 75% regardless the reconstruction set-up studied. The mCT reference noise properties could be achieved using Vision with a scan time reduction (STR) of 1.34 with four iterations and a 440 × 440 matrix size (or STR = 1.89 with a 220 × 220 matrix size) together with a 3D CR improvement of 53% for the 10-mm sphere (24% using 220 × 220).

**Conclusion:**

The Vision exhibited improved NEMA performances compared to mCT. Using the proposed approach, the time acquisition could be divided by almost two, while keeping the same noise properties as that of mCT with a marked improvement of contrast recovery.

## Background

The interest for using silicon photomultipliers (SiPMs) in clinical PET imaging was initially motivated by the known incompatibility of photomultiplier tubes with intense magnetic fields when considering the combination of a PET detector within an MRI system [1]. More specifically, the first released clinical simultaneous PET/MR Biograph mMR (Siemens Healthineers) used avalanche photodiodes (APDs) as photodetectors [2], which prevented the use of time-of-flight (TOF) information due to the low timing resolution. This difficulty was subsequently addressed by the use of SiPMs, which allow a higher gain (at least 10^3^) and a faster rise time (by roughly a factor of three) than APDs [3]. The first TOF PET/MR based on SiPMs technology was released by General Electric (GE Healthcare) with a timing resolution of about 400 ps [4].

Since then, there has been a growing interest to also use SiPMs as photodetectors in PET/CT systems since it paves the way towards a more compact detector, together with an improved timing resolution and a finer decoding scheme of the crystal signal. Indeed, it was shown that highest timing resolution has many advantages in PET/CT among improved signal-to-noise ratio, faster convergence of iterative algorithms, lesser sensitivity to inconsistent or missing data, better imaging properties in cases of very low statistics, and better detectability through the reduction of voxel size [5–7]. The first clinical system with a timing resolution of below 250 ps was recently released by Siemens with the Biograph Vision PET/CT. Two versions are currently available (600 and 450), which differ in the axial field of view (FOV): 26.3 cm for version 600 and 19.7 cm for version 450. The NEMA performance [8] was published for version 600 [9] but not for version 450. The timing resolution for the Vision 600 was measured to be between 210 and 215 ps across a wide range of count rates.

The purpose of this study was, firstly, to derive the NEMA performance of the Vision 450 PET/CT and, secondly, to make a direct comparison of it to the similar (in terms of geometry) mCT with the extended FOV. This study demonstrates the possible improvement achievable when an PMT-based system is replaced with a SiPM-based with the same geometrical properties and the benefits of using an enhanced TOF resolution in the clinical workflow. Two clinical examples are also presented to illustrate the possible improvements in routine clinical practice.

## Methods

### Biograph vision 450 PET/CT

The PET component of the Vision 450 is very similar to that of the version 600. It contains 38 blocks per ring for six rings along the axial FOV. Each block is subdivided into 4 × 2 mini-blocks (four mini-blocks in tangential position for two mini blocks in the axial position), that each contain an array of 5 × 5 LSO crystals of 3.2 × 3.2 × 20 mm^3^. The mini block is coupled to an array of 16 × 16 mm^2^ SiPMs. Table 1 summarizes the main properties of the Vision 450.

**Table 1:** Technical specifications of the PET component for Vision 450 (Vision 600 within brackets) and Biograph mCT

Characteristics	Vision 450 (Vision 600)	Biograph mCT
Crystal dimension	3.2 × 3.2 × 20 mm^3^	4 × 4 × 20 mm^3^
Number of crystals per mini block	5 × 5	NA
Number of mini blocks per block	4 × 2	NA
Number of blocks per ring	38	48
Number of blocks in axial FOV	6 (8)	4
Axial FOV (cm)	19.7 (26.3)	21.6
Image planes	119 (159)	109
Plane spacing (mm)	1.65	2
Bed overlap for step and shoot mode (number of slices)	57 (79)	47
Maximum ring difference	59 (79)	49
Span	17	11
Coincidence time window (ns)	4.7	4.1
Energy window (keV)	435–585	435–650

### NEMA measurements

All measurements were performed following the NEMA procedure including spatial resolution, sensitivity, scatter fraction, noise equivalent count rate (NECR) and accuracy, timing resolution, image quality, and co-registration accuracy. The different experiments were analyzed using the software provided by the manufacturer.

#### Spatial resolution

Because of the expected high spatial resolution of the Vision, a ^22^Na point source (352 kBq) with a dimension (diameter 0.25 mm) suitable to the crystal size was used for all measurements. The source was located in the FOV at given transaxial position (x, y) where x and y, expressed in centimeters, were at the following positions in a given z-plane: (0, 1), (0, 10), and (0, 20) at a z-position of 1/8 axial FOV and ½ axial FOV. The precise position of the source was controlled through a pre-localization step using a specific source L-fixture developed by the manufacturer to ensure that the source was within ± 2 mm for the (x, y) positions and ± 0.25 mm for the z positions. Two million net true coincidences (defined as prompt minus random coincidences) were collected.

Data were reconstructed using a Fourier rebinning combined with a filtered back projection without attenuation, scatter corrections, and using a ramp filter. The voxel size was 0.83 × 0.83 × 0.83 mm^3^ (matrix size 880 × 880 × 237).

#### Sensitivity

A 700-mm long polyethylene tube (inside diameter 1 mm; outside diameter 3 mm) was filled with 4.9 MBq of ^18^F at the start of data acquisition. The source was placed inside the sleeves and positioned at the center and at a 10-cm radial offset. Five data sets corresponding to five specific wall thicknesses were acquired for 300 s each.

#### Scatter fraction and NECR

A cylinder of polyethylene (700-mm long and a diameter of 200 mm) was used. A line source (inside diameter 3 mm, outside diameter 4.8 mm) was inserted axially into the cylinder at a radial position of 45 mm from the phantom center. The initial activity in the line source was 1156 MBq of ^18^F. Thirty-five frames of 240 s were acquired in 11.3 h. The random coincidences were accounted for using the delayed coincidence technique.

#### Timing resolution

The timing resolution was calculated based on the experiment involving the measurement of scatter fraction and NECR. It was estimated as a function of concentration activity following the method proposed by Wang et al. [10].

#### Image quality

The torso-shaped IEC phantom with six coplanar spheres (internal diameters of 10, 13, 17, 22, 28 and 37 mm) was used to evaluate image quality. A central cylindrical insert simulating lung tissue was added to the IEC phantom. The background was filled with a concentration of 5.4 kBq/mL of ^18^F-FDG while the four smallest spheres were filled so that the concentration ratio between the spheres and the background was 4:1 (the two largest spheres were filled with non-radioactive water). Acquisition was performed with the spheres’ center aligned with the axial center of the FOV. The phantom used for the scatter fraction and NECR was placed axially near the IEC phantom to simulate activity outside the FOV. The activity in the line source of this phantom was 153 MBq at the start of the acquisition. The acquisition time was set to 240 s.

Data were reconstructed using the TOF 3D ordinary Poisson ordered subset expectation maximization (3D OP-OSEM) algorithm with point spread function (PSF) recovery and TOF. Two matrix sizes were considered: 220 × 220 (voxel size 3.2 × 3.2 × 1.65 mm^3^) and 440 × 440 (voxel size 1.65 × 1.65 × 1.65 mm^3^). The reconstruction parameters were close to those used in routine clinical practice: four iterations and five subsets without post-filtering. For comparison purposes, data were also reconstructed following the reconstruction parameters matching those used in the seminal work of van Sluis et al. using the Biograph Vision 600 [9] for image quality assessment. The percentage contrast recovery (CR) for each sphere, the percent background variability, the residual errors for attenuation, and scatter corrections were then computed as specified by the NEMA guidelines [8].

#### Co-registration accuracy

A small vial filled with 35 MBq of ^18^F and a CT contrast agent (240 mg/mL) was used for this measurement. The vial was positioned at three transaxial coordinates: (0, 1), (0, 20), and (20, 0) centimeters. A total weight of 115 kg was positioned on the table, and the co-registration accuracy was evaluated at two axial positions (5 cm and 100 cm from the tip of the pallet). A CT scan followed by a 3-min PET acquisition was performed for the six positions considered. PET images were reconstructed using 3D OP-OSEM (10 iterations, 5 subsets, no post-filtering, 440 × 440 matrix size). The co-registration error was subsequently calculated using the software provided by the manufacturer as well as the maximum ratios defined in the NEMA guidelines [8].

### Image quality comparison between vision 450 and mCT

A direct comparison between the Vision and the analog-based mCT with the extended axial FOV [11] was conducted. This comparison will help guide how to sort patients between the two systems and how to maintain consistency in longitudinal studies involving both systems. For this purpose, the IEC NEMA phantom with a sphere-to-background contrast of 4:1 was first acquired on the Vision for 240 s and immediately after on the mCT for 250 s to account for radioactive decay.

The noise level (described hereafter) typically observed using the mCT (with a standard clinical acquisition and reconstruction protocol) was set as the reference value for different numbers of net true coincidences corresponding to acquisition times of 4, 3, 2, 1.5, 1, and 0.5 min. The same number of net true coincidences was chosen for both systems (within 0.01%). The noise level computed using the Vision was matched to the reference noise level (described below) using different reconstruction set-ups (matrix size and reconstruction parameters) to determine the potential reduction of count numbers for the same noise level. Indeed, a similar noise level could be reached with a lower number of counts thanks to the improved time resolution of the Vision. The noise level was computed using the image roughness (IR) as described by Tong et al. [12] based on a single 27-cm^3^-spherical region-of-interest (ROI) so that the same computation could be used with patient data. IR was defined following:

$$ IR=\frac{\sqrt{\frac{1}{N}\sum \limits_{i\in ROI}{\left({\upsilon}_i-\overline{m}\right)}^2}}{\overline{m}} $$

where *N* is the number of voxels in the ROI, *v*_i_ the value of voxel i, and $\overline{m}$ the mean of voxels in the ROI. The image roughness measures the pixel-to-pixel variation and is closely related to the visual perception of noise using a single image. This metric was preferred over background variability (BV) defined in the NEMA standards because BV captures more region-to-region variability, which is better adapted to quantify the variance of background measurement. As noise is not the only parameter relevant in this context, the CR for the hot and cold spheres in 3D considering the entire sphere volume (as opposed to the 2D evaluation used in the NEMA evaluation computed for a 2D cross-section of each sphere) was also computed for each final set-up so that a comparison of contrast could also be achieved. Each voxel intersected by the theoretical surface of the sphere was considered in the computation. The reference reconstruction parameters (mCT) for the 3D OP-OSEM+TOF + PSF used were three iterations and 21 subsets using a matrix size of 200 × 200 (voxel size 4 × 4 × 2 mm^3^). A post-filtering was not applied to ease the interpretation of the results. Additionally, the CR (calculated in 3D) for each sphere size was also derived as a function of IR.

Two clinical cases were also considered to qualitatively illustrate the possible benefits derived from the proposed methodology. As a true whole-body comparison is nearly impossible with ^18^F-FDG given the time difference between the two imaging sessions, two patients were selected with an identical mass, size, and time between injection and PET imaging. The first patient was a 73-year-old (56 kg, 1.56 m) woman evaluated for a breast cancer (injected activity, 173 MBq of ^18^F-FDG; time delay between injection and imaging, 63 min; system used, Biograph mCT; reconstruction parameters, 3D OP-OSEM+PSF + TOF with three iterations, twenty-one subsets, and no post-filtering). The second patient was a 78-year-old (56 kg, 1.57 m) woman evaluated for a recurrence of follicular lymphoma (injected activity, 173 MBq of ^18^F-FDG; time delay between injection and imaging, 60 min; system used, Vision 450; reconstruction parameters, 3D OP-OSEM+PSF + TOF with four iterations, five subsets, and no post-filtering). Vision 450 data were reconstructed by adapting the acquisition time to what was found by using the phantom experiment. Image roughness was calculated in a homogeneous region of the right lobe of the liver for each reconstruction.

The second clinical case referred to a patient treated with ^90^Y-microspheres (2251 MBq of Therapshere®) for a segment VII hepatocellular carcinoma. The radioembolization was for the management of a local recurrence including several satellite nodules. The acquisition time was 30 min for mCT (1 bed step) and Vision (2 beds step of 15 min). The reconstruction parameters were OP-OSEM+PSF + TOF, 3 iterations, 21 subsets, and no post-filtering for mCT (200 × 200 matrix size) and OP-OSEM+PSF + TOF, 4 iterations, 5 subsets, and no post-filtering for Vision as recently suggested in this specific case [13]. The time delay between injection and acquisition was 45.3 h for Vision and 46.1 h for mCT.

## Results

### NEMA measurements

#### Spatial resolution

Table 2 gives the spatial resolution for each source position in terms of FWHM and full width at tenth maximum (FWTM). Results were averaged over two independent measurements.

**Table 2:** Spatial resolution performance

Parameter			
Spatial resolution	Position (cm)	FWHM (mm)	FWTM (mm)
Radial	1	3.5	6.7
	10	4.5	8.2
	20	5.8	10.0
Tangential	1	3.8	7.5
	10	4.0	7.4
	20	3.6	6.7
Axial	1	3.5	6.9
	10	4.2	8.3
	20	4.2	8.9

Source: Na22 source, 0.25-mm diameter

Reconstruction: Fourier rebinning combined with a filtered back projection without attenuation, scatter corrections ,and no post-filtering. The voxel size was 0.83 × 0.83 × 0.83 mm^3^ (matrix size 880 × 880 × 237)

#### Sensitivity

**Fig. 1:**
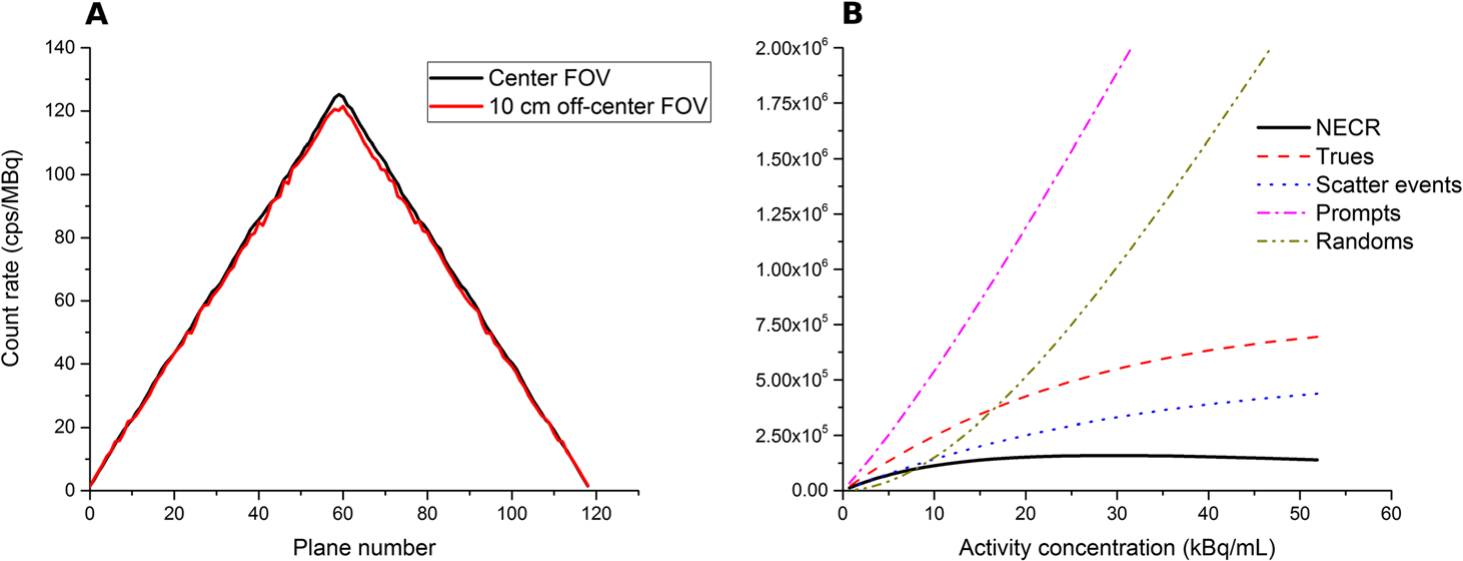
**a** Sensitivity profile at the center and 10 cm off-center of the FOV. **b** NECR and count rates for prompt, true, scatter, and random events

The sensitivity was 9.1 kcps/MBq at the center of the FOV while it was 8.9 kcps/MBq at 10 cm radial distance from the center of the FOV (Fig. 1a).

#### Scatter fraction and NECR

**Fig. 2:**
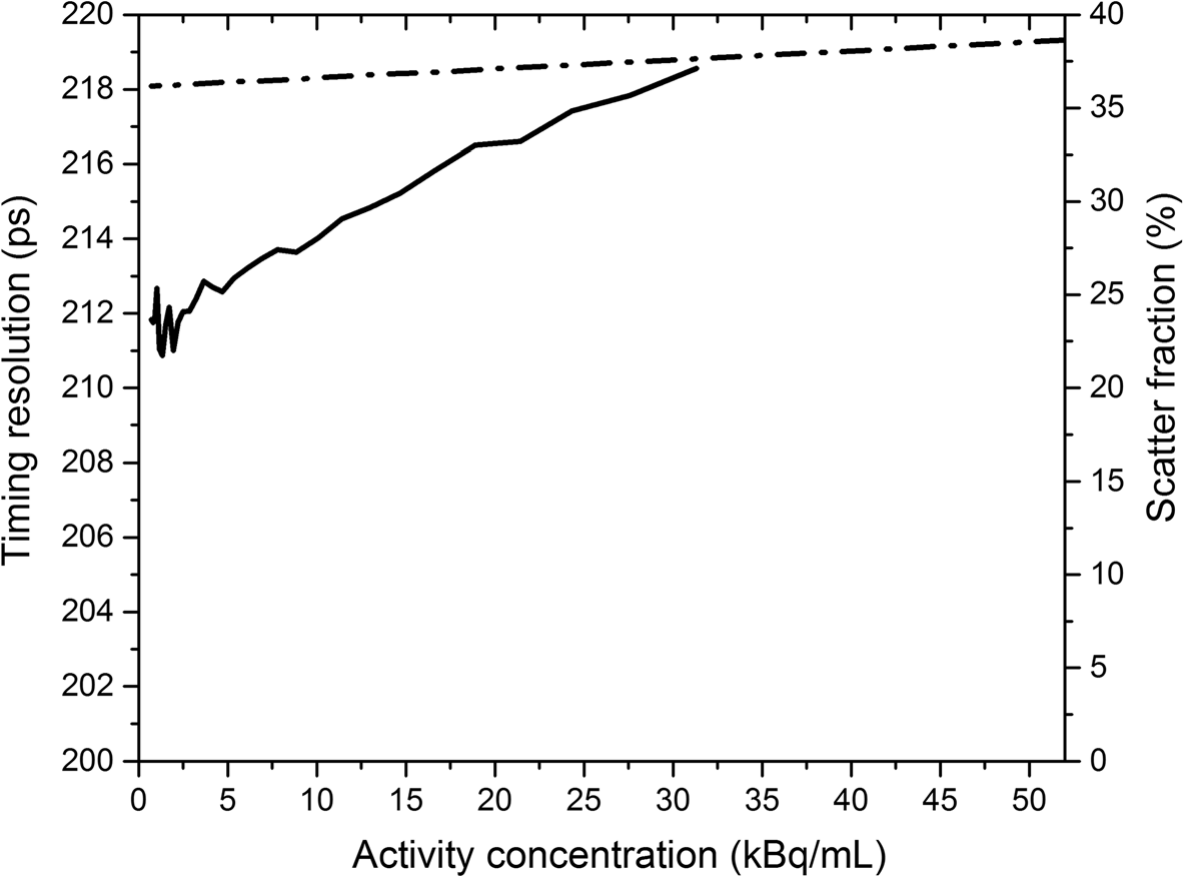
Scatter fraction (dashed-dotted line) and time resolution (solid line) for the activity range studied

Figure 1 b shows the plots of scatter, true, random, prompt coincidences rate, and NECR as a function of activity concentration. Scatter fraction is shown in Fig. 2.

The peak NECR was 160 kcps at 29.3 kBq/mL, and the peak true rate was 694 kcps at 51.9 kBq/mL. The scatter fraction was 37.5% at peak NECR and 36.2% at low activity.

#### Timing resolution

Figure 2 illustrates the dependence of time resolution on activity concentration. The timing resolution was 213 ps at 5.3 kBq/mL and varied at most 3% over the activity range studied.

#### Image quality and co-registration error

Table 3 summarizes the results for the 4:1 contrast ratio and for the two matrix sizes studied (220 × 220 and 440 × 440). The maximum co-registration error was found to be 1.12 mm. All maximum ratios for both PET and CT were less than 0.3 (range [0.16–0.23] for PET and [0.05–0.18] for CT).

**Table 3:** Image quality NEMA results for the 4:1 contrast ratio (3D OP-OSEM+PSF + TOF, 4 iterations, 5 subsets, no post-filtering)

	Contrast recovery (%)		Background variability (%)	
Matrix size	220 × 220	440 × 440	220 × 220	440 × 440
Sphere diameter (mm)				
10	75.4	83.3 (94.4)	5.3	6.3 (9.5)
13	78.4	82.4 (88.0)	4.4	5.0 (7.3)
17	91.0	93.2 (96.5)	3.4	3.7 (5.2)
22	96.3	98.7 (101.8)	2.7	2.7 (3.8)
28	76.7	77.6 (84.6)	2.3	2.3 (3.1)
37	83.2	84.1 (89.9)	1.9	1.9 (2.5)
Average lung residual (%)	5.5	5.5 (3.2)		

Results obtained using 8 iterations, 5 subsets, and no post-filtering are reported within brackets. These values could be directly compared to those reported in Table 3 in [9], for the 10, 13, 17, and 22-mm spheres

### Image quality comparison between vision 450 and mCT

**Fig. 3:**
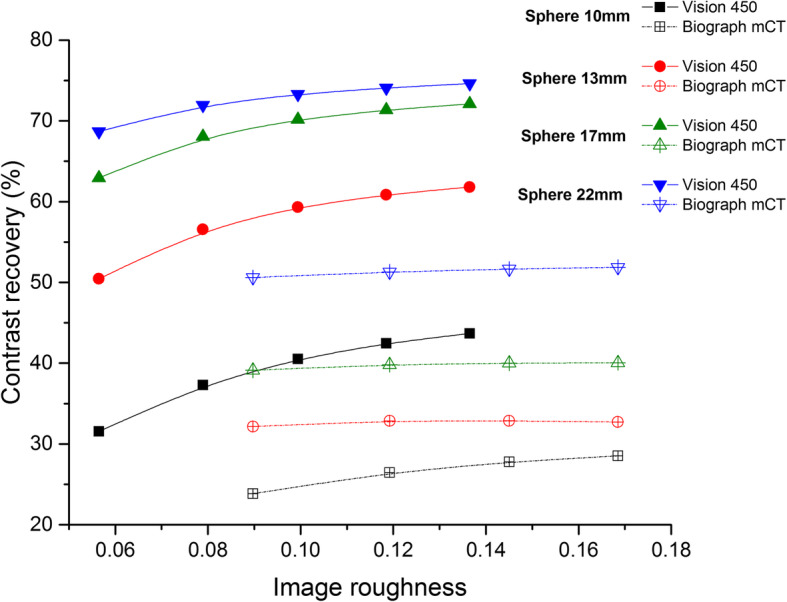
3D contrast recovery computed for Vision (solid line) and mCT (dotted line). Each point is for a specific number of iterations. Datasets from the two scanners have the same number of net trues

**Fig. 4:**
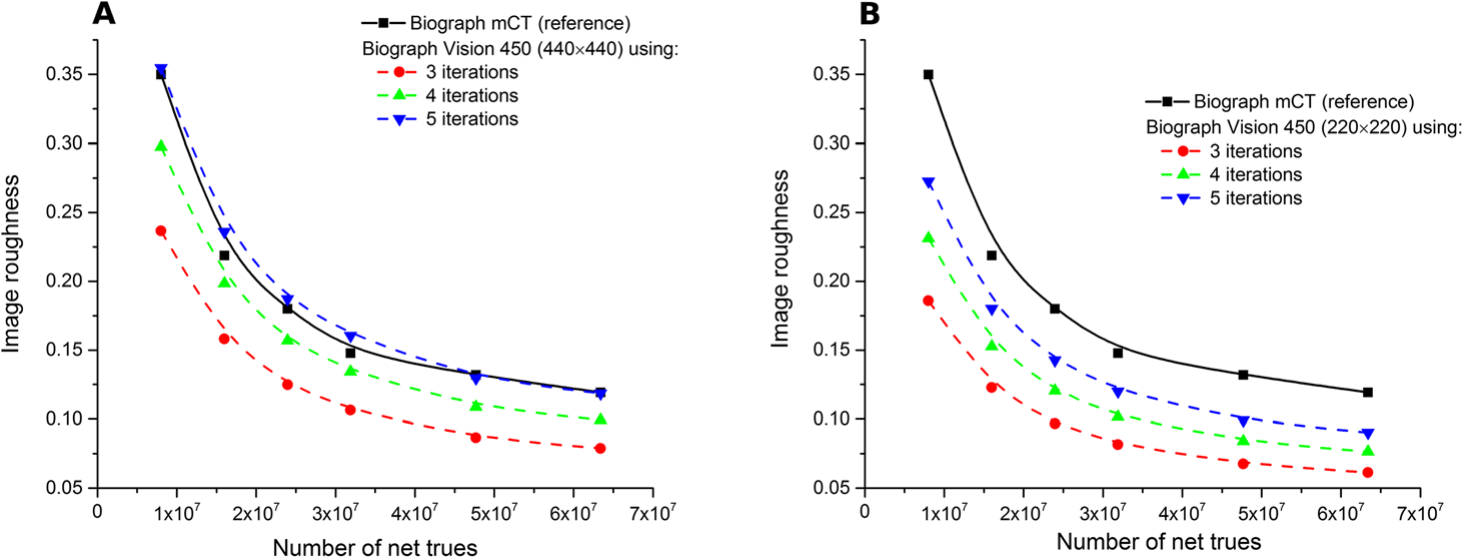
Image roughness as a function of the number of net trues. Data reconstructed from the mCT (set as the reference) using 3 iterations, 21 subsets, and no post-filtering (matrix size 200 × 200). Vision data were reconstructed using 5 subsets, no post-filtering, and between 3 to 5 iterations. **a** 440 × 440 and **b** 220 × 220 matrix size. Data were fitted using a bi-exponential to ease the visual interpretation

Figure 3 shows the 3D contrast recovery as a function of IR for each sphere size for the two systems using the 440 × 440 (Vision) and the 200 × 200 (mCT) matrix size using the same number of net trues (63.4 × 10^6^ coincidences). The comparison to mCT using the 400 × 400 matrix size is provided in supplementary material 1. Figure 4 plots the IR as a function of the number of net trues for the two systems. Both high and low resolution Vision reconstructions show lower noise than the reference low resolution mCT reconstruction, for all iteration numbers considered. As expected, increasing iteration number and lower pixel size resulted in higher noise levels. For comparison purposes, data reconstructed using the 400 × 400 matrix size are presented in supplementary material 2.

**Fig. 5:**
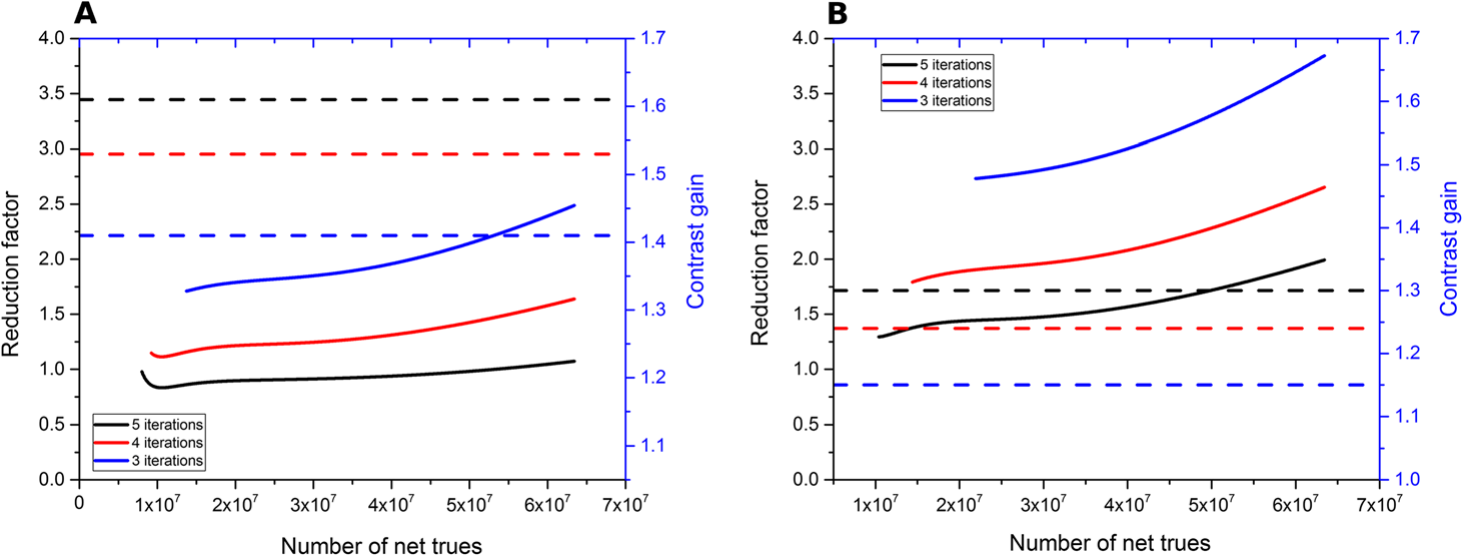
Scan time reduction factor achievable when considering the reconstruction obtained with the mCT as the reference. **a** 440 × 440 and **b** 220 × 220 matrix size for the Vision (using 3 different numbers of iterations). Right y-axis and dashed lines: contrast improvement for the 10-mm sphere (blue, red, and black lines correspond respectively to 3, 4, and 5 iterations)

Given the noise reduction offered by excellent time resolution in the Vision, a reduction of counts (equivalently: acquisition time) can occur. Using the IR as the measure of noise, the possible time reduction factor was computed by fixing the IR of the reference curve and then finding the number of net trues required to reach these IR reference values when reconstructing data using the Vision with different reconstruction parameters (limited to 3, 4, and 5 iterations and 5 subsets). Each curve was arbitrarily fitted with a bi-exponential function to increase the precision (or number of points) of the reduction factor computation and ease the visualization. Figure 5 shows the results for the 440 × 440 and the 220 × 220 matrix size. The mean reduction factors for each case are given in Tables 4 and 5. They are computed only for a number of net trues less than 35 × 10^6^, as this is mainly the condition encountered in clinical routine practice [14]. The gain in terms of contrast recovery was also reported. This last one was derived for each number of iterations from the reconstruction with the highest number of net trues, as the CR was not supposed to change with the number of net trues. This assessment was conducted using the 200 × 200 matrix size from the mCT as reference (additional results when considering the 400 × 400 matrix size are presented in supplementary material 3).

**Table 4:** Scan time reduction factor and CR gain for different numbers of iterations (Vision with 440 × 440 matrix size) when compared to the reference values of mCT

		Contrast recovery gain (%)					
Number of iterations	Mean reduction factor	10-mm sphere	13-mm sphere	17-mm sphere	22-mm sphere	28-mm sphere	37-mm sphere
3	1.79	+ 41%	+ 72%	+ 71%	+ 40%	+ 14%	+ 9%
4	1.34	+ 53%	+ 81%	+ 76%	+ 43%	+ 20%	+ 14%
5	0.95	+ 61%	+ 85%	+ 79%	+ 44%	+ 25%	+ 17%

**Table 5:** Reduction factor and CR gain for different numbers of iterations (Vision with 220 × 220 matrix size) when compared to the reference values of mCT

		Contrast recovery gain (%)					
Number of iterations	Mean reduction factor	10-mm sphere	13-mm sphere	17-mm sphere	22-mm sphere	28-mm sphere	37-mm sphere
3	2.77	+ 15%	+ 37%	+ 40%	+ 14%	+ 1%	+ 0%
4	1.89	+ 24%	+ 43%	+ 44%	+ 16%	+ 7%	+ 4%
5	1.44	+ 30%	+ 46%	+ 46%	+ 17%	+ 10%	+ 6%

**Fig. 6:**
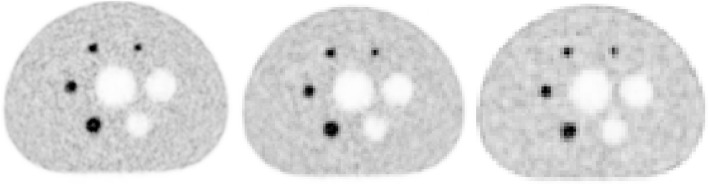
Reconstructed images of the IEC phantom with the same image roughness (IR = 0.15). The reference was for the mCT using 3 iterations, 21 subsets, no post-filtering, 200 × 200 matrix size, and 3.2 × 10^7^ net trues (equivalent to a time acquisition of 120 s). Left: Vision using 4 iterations, 5 subsets, no post-filtering, 440 × 440 matrix size, and 2.4 × 10^7^ net trues (reduction factor = 1.34, time acquisition = 89 s); middle: Vision using 4 iterations, 5 subsets, no post-filtering, 220 × 220 matrix size, and 1.7 × 10^7^ net trues (reduction factor = 1.89, time acquisition = 63 s); right: mCT (reference). Gray scale level is identical for all images. Contrast improvement for the different spheres, as compared to the reference, is listed in Tables 4 and 5.

**Fig. 7:**
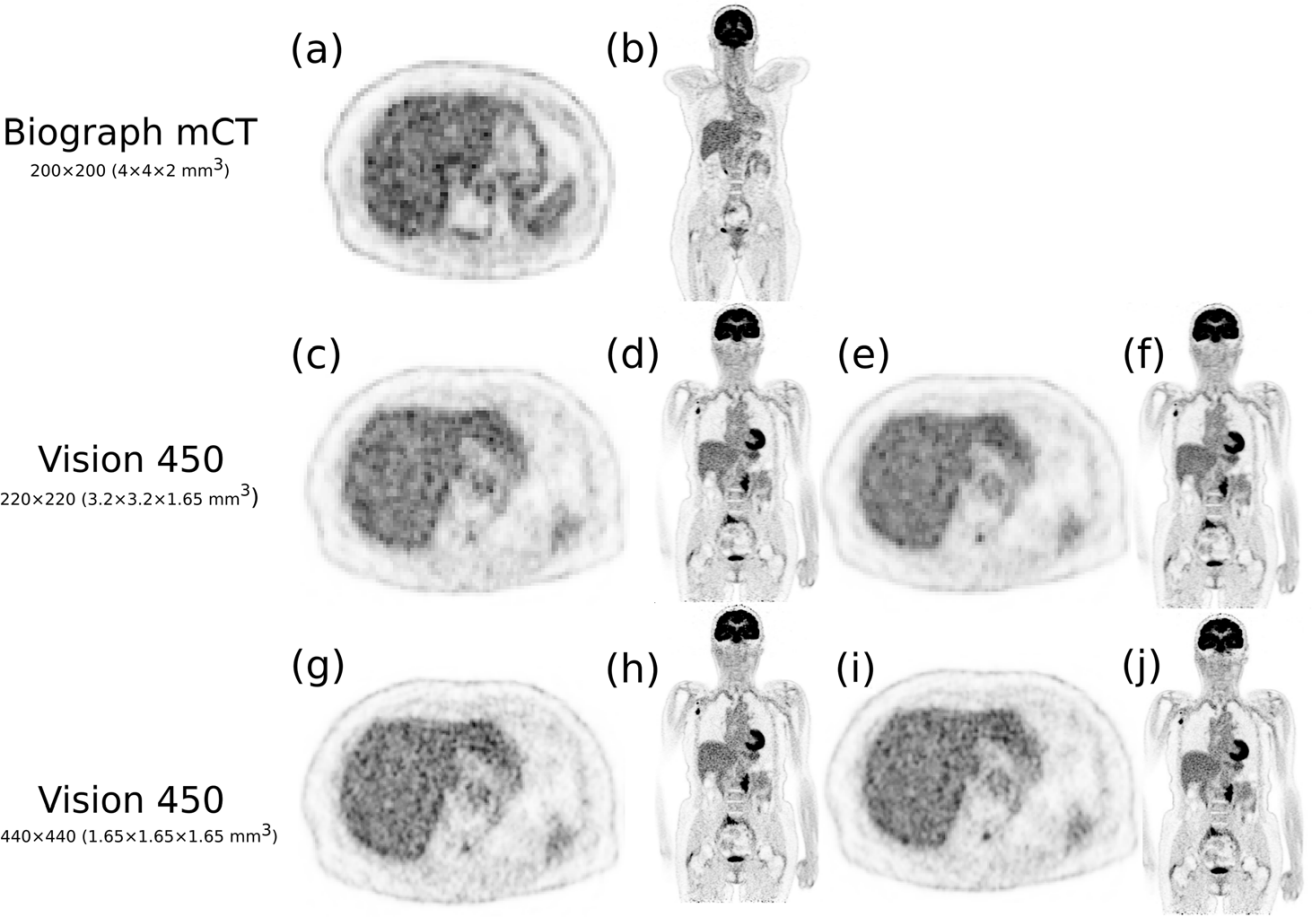
^18^F-FDG-PET evaluated for two patients sharing the same morphological properties and injected activity (173 MBq): breast cancer imaged on the Biograph mCT (**a**, **b**) and follicular lymphoma imaged on the Biograph Vision 450 (**c**, **d**, **e**, **f**, **g**, **h**, **i**, **j**). Reconstruction parameters for mCT was OP-OSEM+PSF + TOF, 3 iterations, 21 subsets, and no post-filtering (200 × 200 matrix size) and OP-OSEM+PSF + TOF, 4 iterations, 5 subsets, and no post-filtering for the Biograph Vision 450. The reduction factor as compared with time acquisition used for mCT was 1.89 for the 220 × 220 matrix size (**c**, **d**) and 1.34 for the 440 × 440 matrix size (**g**, **h**). Data reconstructed using a time per step identical to those of Biograph mCT are presented in (**e**, **f**) for the 220 × 220 matrix size and (**i**, **j**) for the 440 × 440 matrix size. The IR computed on the right liver lobe was almost identical: 0.176 (**a**, **b**); 0.178 (**c**, **d**); and 0.183 (**g**, **h**). The IR for (**e**, **f**) was 0.118 and 0.142 for (**i**, **j**). Grey scale is identical for all images

**Fig. 8:**
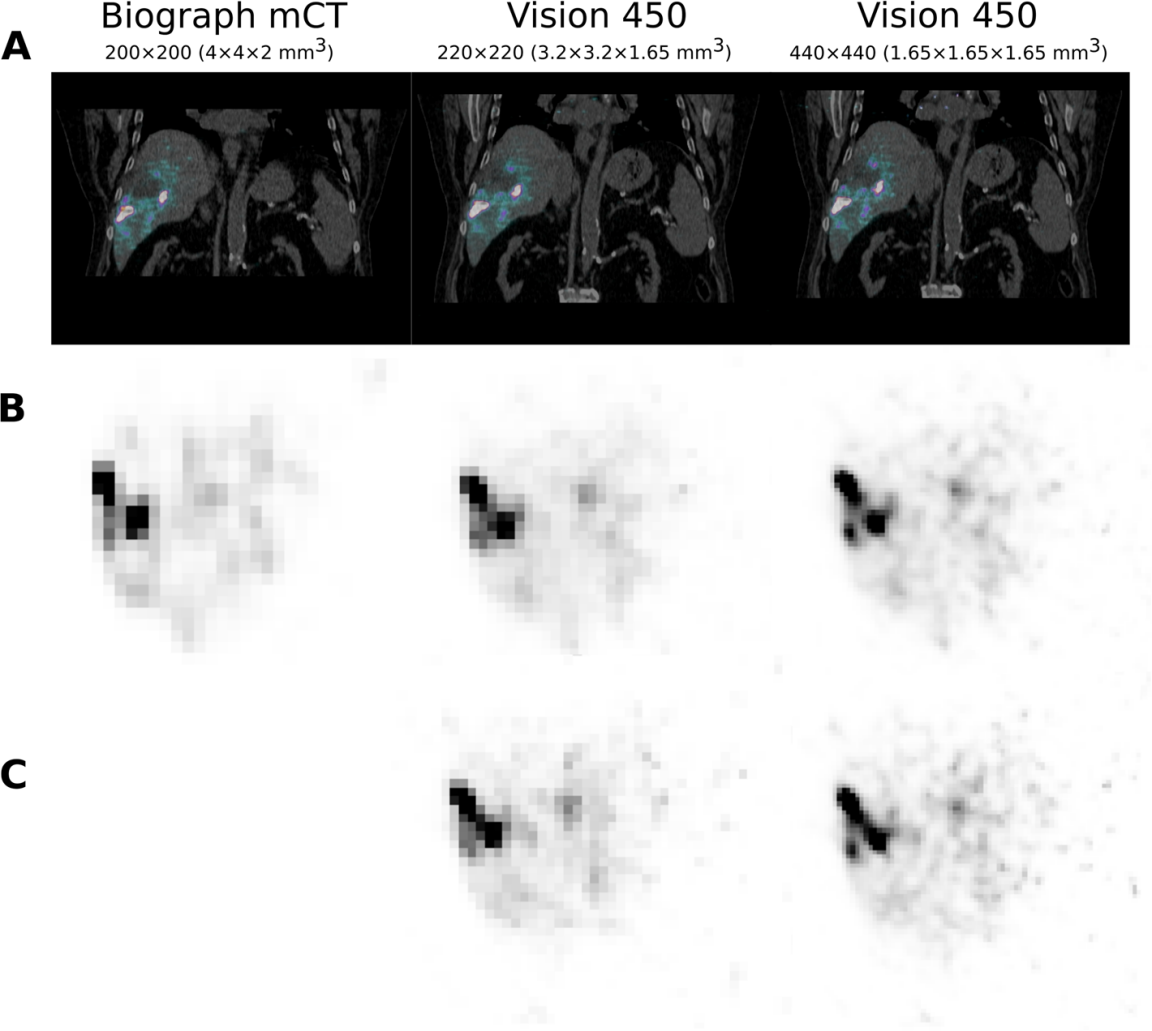
Patient with segment VII hepatocellular carcinoma. The radioembolization was for the management of a local recurrence including several satellite nodules. The acquisition time was 30 min for mCT and Vision (injected activity 2251 MBq of ^90^Y-microspheres). The reconstruction parameters were OP-OSEM+PSF + TOF, 3 iterations, 21 subsets, and no post-filtering for mCT (200 × 200 matrix size) and OP-OSEM+PSF + TOF, 4 iterations, 5 subsets, and no post-filtering for Vision. Biograph mCT and Vision 450 images were co-registered using rigid transformation. **a** Fused coronal slice, **b** selected transaxial slice using full time acquisition for each case, **c** selected transaxial slice using 15 min 52 s (reduction factor 1.89) for Vision using the 220 × 220 matrix size (middle) and 22 min 23 s (reduction factor 1.34) for Vision using the 440 × 440 matrix size. Grey scale was identical

Figure 6 illustrates the effect of reducing the time acquisition using the IEC phantom when considering the image reconstructed with the mCT as the reference. In this case, the target IR was set to 0.15 (equivalent to a time acquisition of 120 s for the mCT). The reduction factor was defined as the reduction in time that would produce an image matching the target IR = 0.15. The reconstructed images for Vision using the 440 × 440 and the 220 × 220 matrix size (four iterations and five subsets for both) are presented and corresponded to a reduction time factor of, respectively, 1.34 (acquisition time 89 s) and 1.89 (acquisition time 63 s). The clinical case using ^18^F-FDG for two patients injected with the same activity and sharing the same mass and size is presented in Fig. 7. The reduction factor derived previously was applied to reconstructed data acquired with the Vision. Given that the reference acquisition time per step using the Biograph mCT was 120 s, data were reconstructed using 220 × 220 matrix size and 63 s per step (which represented a scan time reduction of 1.89) and 89 s per step (which represented a scan time reduction of 1.34) using 440 × 440 matrix size. The IR computed in the right lobe liver was almost identical for all reconstruction: 0.176 for the patient imaged with mCT (120 s per step), 0.178 and 0.183 for the patient imaged with Vision (respectively 220 × 220 and 440 × 440 matrix size). The marked improvement of IR when using a matched time acquisition between mCT and Vision is also highlighted as expected for either the 220 × 220 or 440 × 440 matrix size. Conversely, a true comparison (that is, same patient imaged using Vision 450 and immediately after, within 5 min, using between Biograph mCT) is presented in Fig. 8 for the patient injected with ^90^Y-microspheres. In this specific case, the reconstruction set-up was the one suggested recently in [13].

## Discussion

### NEMA performances

NEMA performances of the Vision 450 were compatible to those published, for the Vision 600, in the work of van Sluis et al. [9]. Specifically, spatial resolution measurements were in line, within a few percent, with those reported for the Vision 600. Sensitivity was around 9.1 kcps/MBq at the center of the FOV, which was 45% lower than that of the Vision 600 (16.4 kcps/MBq) and 6% lower than the value reported for the mCT (9.7 kcps/MBq) [11]. This was most likely due to the difference in the axial FOV between the different systems: 26.3 cm and 21.8 cm for the Vision 600 and mCT, respectively, and 19.7 cm for the Vision 450. It is interesting to note that the experimental ratio of sensitivity between Vision 600 and Vision 450 is well in accordance with the ratio calculated using the model proposed by Eriksson and colleagues [15] (experimental 1.80 vs model 1.84).

Accordingly, the peak NECR was found to be 48% lower than the value reported for the Vision 600 (11% for the mCT) while the scatter fraction remained the same (37.5% for Vision 450 vs 37% for Vision 600) at low activity. The time resolution reported with the Vision 600 [9] was confirmed in this study as 213 ps at 5 kBq/mL, very close to the 210 ps of the previous publication. The image quality measurement as proposed by the NEMA standard depended heavily on the reconstruction algorithm used. The 2D contrast recovery was 75% and 83% for the 220 × 220 and 440 × 440 matrices, respectively, which were higher than the 52% and 65% reported in [11]. The reconstruction parameters used were identical to those used in [11]. This highlights the benefit of the improved time resolution acquired with the smaller crystal size of the Vision 450 as compared to those embedded in the Biograph mCT. Additionally, the 2D contrast recovery for the 10-mm-diameter sphere was found to be roughly the same (94.4% vs 93.1%) as results obtained with the Vision 600 [9]. Figure 3 showed that the marked 3D contrast recovery improved together with a concomitant significant noise reduction, as measured by the image roughness. This was a direct consequence of a better time resolution, as described previously [7].

### Clinical benefits achievable in routine practice

The installation of a SiPM-based system in a department often raises several questions about the choice of acquisition and reconstruction parameters to be used, especially when the system replaces a PMT-based system with an identical geometry and hence a very similar sensitivity. In this work, a simple method was proposed to give an idea of what the reduction factor could be, given a targeted reference noise level defined using standard parameters of a PMT-based system. Specifically, it was shown that a noise level set using standard reconstruction parameters of the mCT could be obtained with a reduction factor of 1.34 (four iterations, five subsets, and a 440 × 440 image matrix) or of 1.89 (four iterations, five subsets, and a 220 × 220 image matrix). Each reduction factor resulted in significant 3D contrast recovery improvement. For example, the 10-mm-diameter sphere benefited from an increase of + 53% (respectively, + 24%) when using the 440 × 440 matrix and, respectively, the 220 × 220 matrix (Tables 4 and 5). Hence, it allowed for the reduction of counts (and then significantly the acquisition time), which kept the noise level the same as that of the mCT yet still improved contrast recovery. This reduction of acquisition time can be translated to a reduction of injected activity given the linear behavior of count with activity concentration when considering clinical activity range (supplementary material 4). A larger image matrix size (440 × 440, 1.65 × 1.65 × 1.65 mm^3^ voxel size) could routinely be used while keeping the noise level compatible in order to benefit from a high 3D contrast recovery and hence a better detectability. It should be also emphasized that there was an almost strict equivalence in terms of noise between the mCT (three iterations, 21 subsets, 200 × 200) and the Vision (five iterations, five subsets, 440 × 440) regardless of the number of net trues for the count rate used in this work (Fig. 5). This last finding highlights the possibility to maximize the detectability by using more iterations and the smallest voxel size while keeping the same noise level. It is worth noting that the approach developed in this study could also be conducted using post-filtering, as is done in routine clinical conditions. However, post-filtering was not applied in this study. Adding an extra-parameter (post-filtering) would have made the analysis more complex (more possibilities to be taken into account) without adding significant valuable information.

The results obtained using the NEMA phantom paved the way to a new era for oncological exams. Apart from the reduction factor using the 440 × 440 matrix size presented above, it is possible to reach a factor of three (Table 5) using the 220 × 220 matrix size and still gain in contrast recovery if users are more interested in reducing injected activity or in speeding up the acquisition time for pediatric studies, for example. In other words, while a larger pixel size (4 × 4 × 2 mm^3^ for 200 × 200) had to be chosen for the mCT as a clinical standard to obtain a low noise level, with Vision 450, a full resolution (1.65 × 1.65 × 1.65 mm^3^ for 440 × 440) could be used because of the noise reduction due to better TOF performance. Of note, these findings are roughly in line with those recently reported by Gnesin and colleagues [16]. They found a reduction factor of 3.12 when comparing mCT (512 × 512, three iterations, 21 subsets, and no post-filtering) to the Vision 600 (512 × 512, four iterations, five subsets, and no post-filtering) for a coefficient of variation of 15%. In the same settings (apart from the mCT matrix size used in this study 400 × 400), a reduction factor of 2.88 was reported for an IR of 16.5%.

Obviously, this conclusion cannot be seen as a general rule valid for any type of patient, since only one phantom with specific geometrical properties was used. In that respect, the reduction factor may be different for heavier patients, who are more likely to benefit from the improved time resolution [17]. It is also worth noting that the reduction factors obtained in this work are only valid for the specific count rate for which they were calculated. The expectable dependency with count rate could be derived through the use of NECR curves which are available for both system. Additionally, while IR could be seen as a surrogate of noise and visual perception, the image texture may not be exactly the same when comparing two images with different voxel size (Fig. 6) although the IR is identical. Yet, more studies need to be conducted to consider different types of geometry using phantom or clinical data. The difficulty of using the same clinical data extracted from two different systems can be partially overcome with a random dual-imaging protocol as performed recently for the Vision 600 [18]. It is also possible to focus only on lesion detectability. A few interesting results were recently highlighted when comparing mCT and Vision 600 using a dedicated torso phantom [19]. That study found a possible reduction factor of between four and six for a nearly matched voxel size. Yet, optimization in this study was done on ^18^F and with count statistics typical of ^18^F-FDG oncology imaging. Such optimization might need to be repeated for tracers that exhibit different statistics and contrast or organ distribution.

## Conclusion

The Vision 450 exhibited very similar results as the Vision 600 in terms of spatial resolution, time resolution, and co-registration accuracy. It was shown that a significant reduction factor (≈ 2) for time acquisition could be achieved using approximately the same matrix size while significantly improving the contrast recovery. The use of a larger matrix was also routinely feasible at a same noise level as that of the mCT, with a reduction factor of 1.34 and a marked improvement of contrast recovery.

## Supplementary information

**Additional file 1:.** Supplemental figure 1. 3D contrast recovery computed for Vision (solid line) and mCT (dotted line). Data were reconstructed using 4 iterations, 5 subsets, no post-filtering (matrix size: 440×440) for the Vision and 3 iterations, 21 subsets, no post-filtering (matrix size: 400×400) for the mCT. Datasets from the two scanners have the same number of net trues. Supplemental figure 2. Image roughness as a function of the number of net trues. Data reconstructed from the Biograph mCT using 3 iterations, 21 subsets, no post-filtering and a 400×400 matrix size (set as the reference). Biograph Vision 450 data were reconstructed using the 440×440 matrix size, 5 subsets, no post-filtering and between 3 to 5 iterations. Supplemental figure 3. Scan time reduction factor achievable when considering the reconstruction obtained with the Biograph mCT (3 iterations, 21 subsets, no post-filtering and 400×400 matrix size) as the reference using the 440×440 matrix size for the Biograph Vision 450 (different number of iterations of the OP-OSEM+TOF+PSF algorithm attached to the Biograph Vision 450 were considered). Right y-axis and dashed lines: contrast improvement for the 10-mm sphere (blue, red and dark line colors correspond respectively to 3, 4 and 5 iterations). Supplemental figure 4. Net count as a function of activity concentration for 18F-FDG clinical activity. NEC dependence with activity concentration was fitted using a linear model.

## Data Availability

The datasets generated during and/or analyzed during the current study are available from the corresponding author upon reasonable request.
