# Supplementary material for: From a PMT-based to a SiPM-based PET system: a study to define matched acquisition/reconstruction parameters and NEMA performance of the Biograph Vision 450
Source: EJNMMI Phys. 2020 Sep 3;7:55. doi: 10.1186/s40658-020-00323-w (PMC7471223; doi:10.1186/s40658-020-00323-w)
Supplement: Supplementary file 1 — Additional file 1:. Supplemental figure 1. 3D contrast recovery computed for Vision (solid line) and mCT (dotted line). Data were reconstructed using 4 iterations, 5 subsets, no post-filtering (matrix size: 440×440) for the Vision and 3 iterations, 21 subsets, no post-filtering (matrix size: 400×400) for the mCT. Datasets from the two scanners have the same number of net trues. Supplemental figure 2. Image roughness as a function of the number of net trues. Data reconstructed from the Biograph mCT using 3 iterations, 21 subsets, no post-filtering and a 400×400 matrix size (set as the reference). Biograph Vision 450 data were reconstructed using the 440×440 matrix size, 5 subsets, no post-filtering and between 3 to 5 iterations. Supplemental figure 3. Scan time reduction factor achievable when considering the reconstruction obtained with the Biograph mCT (3 iterations, 21 subsets, no post-filtering and 400×400 matrix size) as the reference using the 440×440 matrix size for the Biograph Vision 450 (different number of iterations of the OP-OSEM+TOF+PSF algorithm attached to the Biograph Vision 450 were considered). Right y-axis and dashed lines: contrast improvement for the 10-mm sphere (blue, red and dark line colors correspond respectively to 3, 4 and 5 iterations). Supplemental figure 4. Net count as a function of activity concentration for 18F-FDG clinical activity. NEC dependence with activity concentration was fitted using a linear model. [file 40658_2020_323_MOESM1_ESM.pdf]

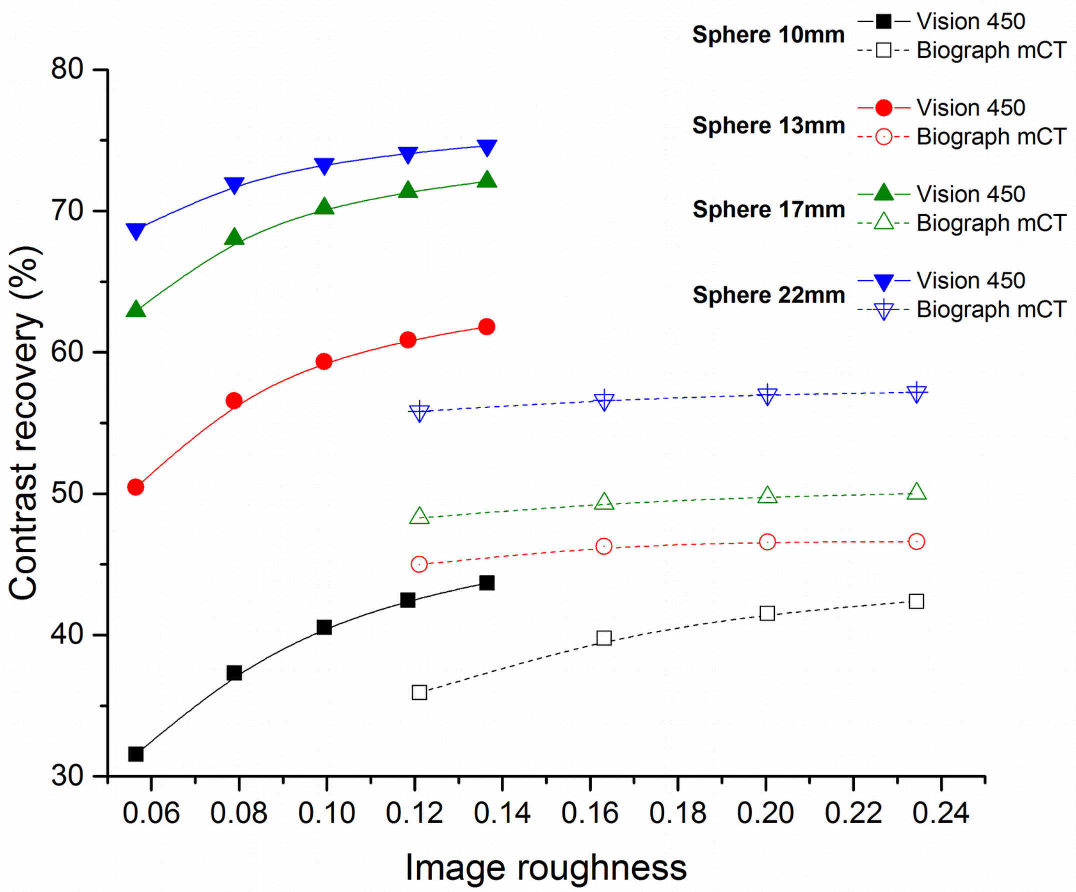

Supplemental figure 1. 3D contrast recovery computed for Vision (solid line) and mCT (dotted line). Data were reconstructed using 4 iterations, 5 subsets, no post-filtering (matrix size: 440×440) for the Vision and 3 iterations, 21 subsets, no post-filtering (matrix size: 400×400) for the mCT. Datasets from the two scanners have the same number of net trues.

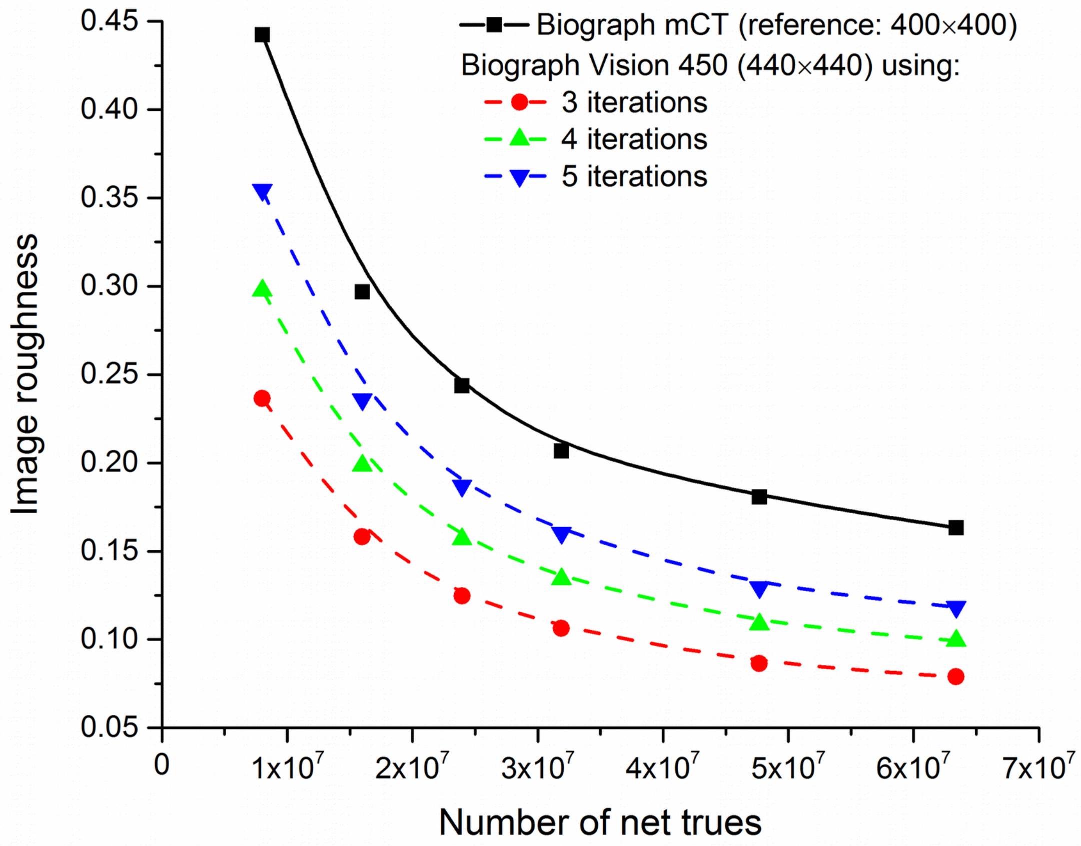

Supplemental figure 2. Image roughness as a function of the number of net trues. Data reconstructed from the Biograph mCT using 3 iterations, 21 subsets, no post-filtering and a 400×400 matrix size (set as the reference). Biograph Vision 450 data were reconstructed using the 440×440 matrix size, 5 subsets, no post-filtering and between 3 to 5 iterations.

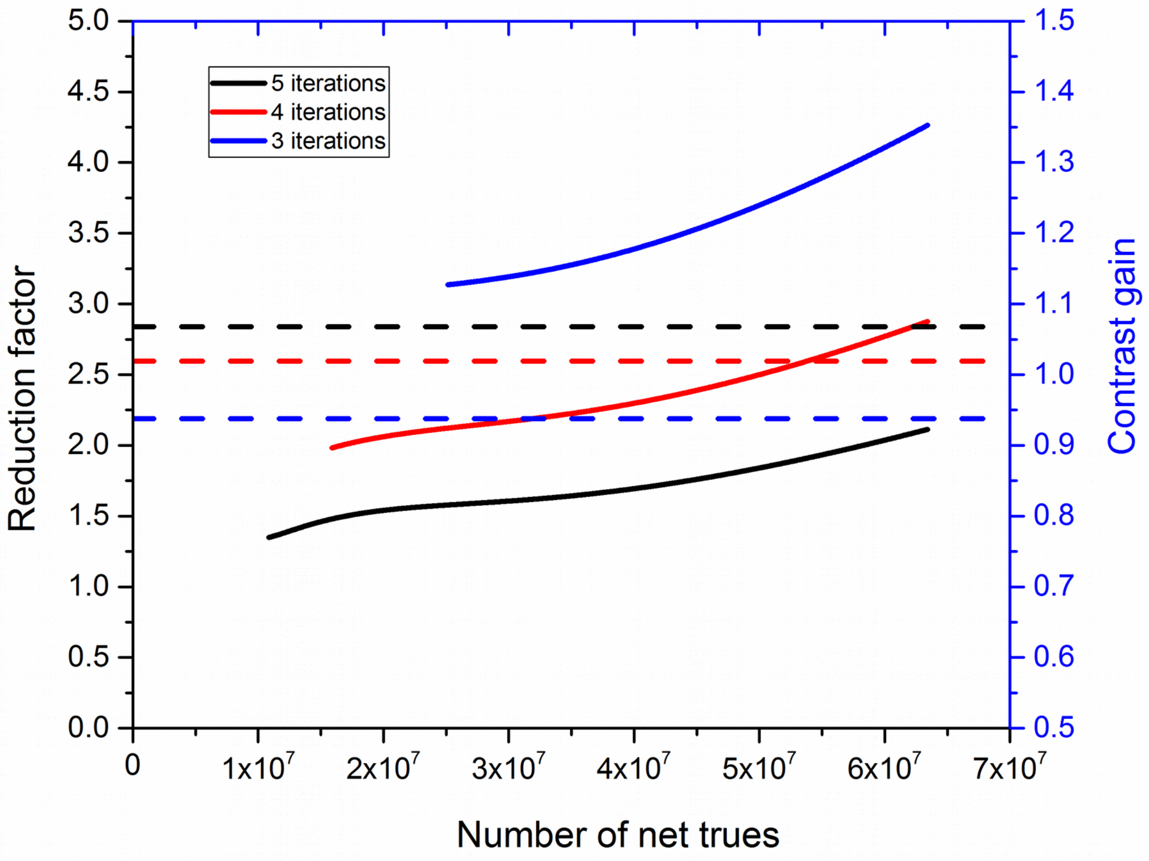

Supplemental figure 3. Scan time reduction factor achievable when considering the reconstruction obtained with the Biograph mCT (3 iterations, 21 subsets, no post-filtering and 400×400 matrix size) as the reference using the 440×440 matrix size for the Biograph Vision 450 (different number of iterations of the OP-OSEM+TOF+PSF algorithm attached to the Biograph Vision 450 were considered). Right y-axis and dashed lines: contrast improvement for the 10-mm sphere (blue, red and dark line colors correspond respectively to 3, 4 and 5 iterations).

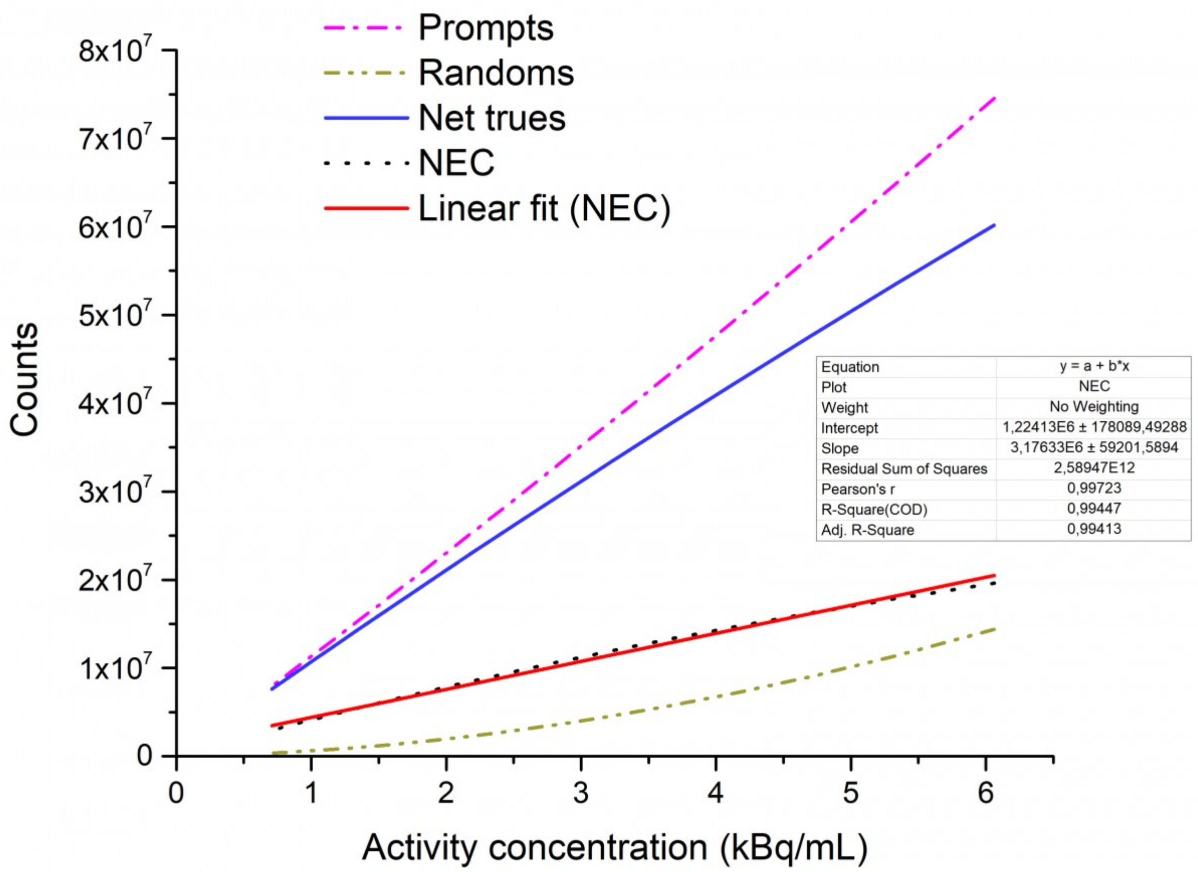

Supplemental figure 4. Net count as a function of activity concentration for ^{18}F-FDG clinical activity. NEC dependence with activity concentration was fitted using a linear model.
